# *KRAS* mutant lung cancer: progress thus far on an elusive therapeutic target

**DOI:** 10.1186/s40169-015-0075-0

**Published:** 2015-12-14

**Authors:** Saveri Bhattacharya, Mark A. Socinski, Timothy F. Burns

**Affiliations:** University of Pittsburgh Cancer Institute, 5150 Centre Avenue, Room 461, Pittsburgh, PA 15232 USA; Medicine and Cardiothoracic Surgery, University of Pittsburgh Cancer Institute, 5150 Centre Avenue, Room 556, Pittsburgh, PA 15232 USA; University of Pittsburgh Cancer Institute, 5117 Centre Avenue, Office: Suite 2.18e, Pittsburgh, PA 15232 USA

**Keywords:** *KRAS* mutant NSCLC, MAPK pathway, Targeted therapy

## Abstract

The *KRAS* mutation remains the most common driver mutation in patients with non-small cell lung cancer (NSCLC) and confers a poor prognosis. Thus far, efforts to target this mutation over the last two decades have been unsuccessful. Over the past 5 years, many efforts to develop drugs that target the RAS-RAF-MEK-ERK (MAPK) pathway have resulted in enhanced understanding of the *KRAS* mutant NSCLC and have provided optimism that this disease can be targeted.

## Introduction

Lung cancer is the leading cause of cancer mortality in the United States [1]. Currently, the 5-year survival remains an abysmal 17 %. In 2015, in the US alone, an estimated 158,040 patients are expected to die of lung cancer accounting for 27 % of all cancer deaths and accounts for more deaths than prostate, colorectal and breast cancers combined [1]. The advances made in the last decade have revealed that non-small cell lung cancer (NSCLC) is not a single cancer, but rather a collection of molecularly defined neoplasms with distinct biology and clinical outcomes. This principle is supported by the seminal finding that sensitizing epidermal growth factor receptor (*EGFR*) mutations are present in about 17 % of lung cancers and are targetable with the FDA approved EGFR tyrosine kinase inhibitors (TKIs) (erlotinib, gefitinib and afatinib) [2, 3]. Additionally, translocations involving the anaplastic lymphoma kinase (ALK) are found in 7 % of adenocarcinomas of the lung and can be targeted by several ALK TKIs (crizotinib and ceritinib) [2, 3]. However, currently there are no FDA approved drugs that target the most common driver oncogenic driver, mutant *KRAS* [4].

The *KRAS* mutation is present in approximately 25 % of patients with NSCLC (mostly adenocarcinoma) and was first discovered more than 3 decades ago [5]. This mutation confers a poor prognosis in the metastatic setting, and a high risk of cancer recurrence as seen in several studies [6–9]. It is mutated in one-third of all cancers including colon cancer and pancreatic cancer. In addition, the three human RAS genes (*NRAS, HRAS, KRAS*) have been identified and mutations in these three isoforms have been seen throughout human cancers [5].

**Fig. 1:**
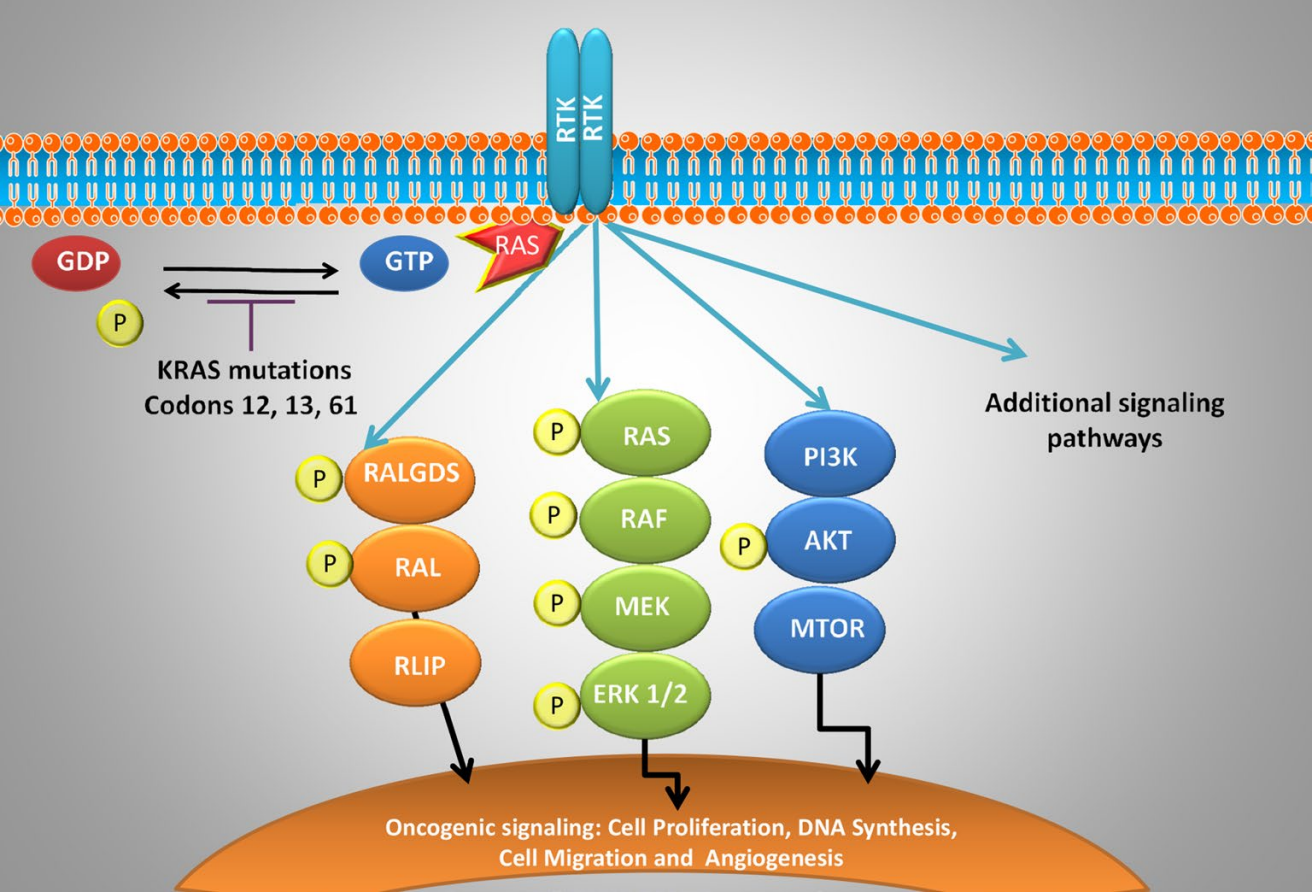
The RAS-RAF-MEK-ERK Signaling Cascade in NSCLC. In a normal cell, the activation of the KRAS protein through binding of GTP and translocation to the plasma membrane is a tightly controlled process. However, in NSCLC, the KRAS protein is often mutated at codons 12, 13, and 61 leading to the inactivation of its intrinsic GTPase activity resulting in constitutive activation of KRAS. Mutant KRAS can then promote tumorigenesis through multiple downstream signaling pathways

In lung cancer, *KRAS* mutations occur frequently at codons 12 and 13 and less frequently at codon 61 [10]. The most frequently observed mutation in lung cancer is G12C and is associated with exposure to tobacco [11]. The mutation G12C accounts for 40 % of total mutations, followed by G12V (22 %) and G12D (16 %) [12, 13]. Interestingly, *KRAS* mutations at G12C and G12V have a worse clinical outcome possibly due to their ability to engage in multiple downstream effectors including the RAL pathway [14]. Conversely, the G12D mutant protein predominantly activates the RAF/MAPK and PI3K pathways [14] (Fig. 1). Finally, codon 61 mutant are more severely deficient in intrinsic GTPase activity and may therefore have increased activity compared to alterations at codons 12 and 13 [15].

Regardless of the site of the mutation, these mutations result in loss of GTPase activity making this oncoprotein constitutively active and leading to activation of a series of downstream pathways including the RAF-MEK-ERK (MAPK) signaling pathway and AKT-PI3K-MTOR pathway (Fig. 1). Thus far, efforts to inhibit *KRAS* have focused primarily on targeting the RAS-RAF-MEK-ERK (MAPK) signaling pathway.

## RAS-RAF-MEK-ERK pathway

In the RAS signaling cascade, the binding of either GTP or GDP to RAS serves as the “on” or “off” switch for RAS signaling respectively. In the normal cell, RAS is GDP bound and is inactive unless an extracellular stimuli causes formation of an active GTP-bound molecule. RAS is subsequently inactivated through hydrolysis of its GTP to GDP primarily through the function of GTPase-activating proteins (GAPs). Upon mutation, its intrinsic GTPase activity is lost and GAPs are unable to bind RAS resulting in RAS primarily bound to GTP and therefore constitutively activated [16]. *KRAS* mutations in lung cancer occur primarily at codon 12 or 13, making the protein GAP insensitive and constitutively GTP bound leading to the activation of downstream effectors. It then drives oncogenesis through a multitude of effectors and downstream signaling pathways to promote tumor growth. These signaling pathways include RAF (MAP kinase pathway), PI3K (AKT/MTOR pathway), ERK, RLIP and RALGDS as seen in Fig. 1. The unregulated signaling of RAS in these pathways thereby leads to increased proliferation, decreased apoptosis, disrupted cellular metabolism, and increased angiogenesis which in turn leads to tumor cell proliferation [16].

The RAF, MEK, ERK, PI3K, AKT, MTOR, and RALGDS pathways are targets for drug development. However, an understanding of the nature of these pathways is paramount before designing therapeutic strategies. For example, activated RAF phosphorylates and activates the kinase MEK, which then phosphorylates and activates the ERK kinase. Upon activation, ERK phosphorylates a number of substrates including kinases and transcription factors that mediate entry and progression through the cell cycle, inhibition of differentiation, protein translation and suppression of apoptosis [16]. Despite understanding the underlying cascade for the RAF/MEK/ERK pathway, it is still unclear what node is the most efficacious to target clinically. Therefore, not only is an understanding of the critical signaling pathways downstream of *KRAS* required but also the knowledge of which node to target within in these essential pathways. Finally, it is clear that an understanding of the critical pathways for each *KRAS* mutant codon [14] and possibly mutational subset (*KRAS/STK11* or *KRAS/TP53*) [17, 18] will be required to target *KRAS* mutant NSCLC.

Over the last two decades, a variety of strategies have been developed and tested to target oncogenic *KRAS* signaling. These include the development of direct inhibitors of the KRAS protein, use of RNA interference strategies, development of inhibitors which prevent localization of RAS to the plasma membrane, and pharmacologic targeting of its downstream effectors. It remains to be seen whether any of these strategies will produce a significant clinical benefit. Both the past and present early clinical trial efforts to test these clinical trial strategies are described.

## Previous efforts to target *KRAS* mutant NSCLC in the clinic

### Cytotoxic chemotherapy

Although platinum based chemotherapy remains the standard of care in the first line adjuvant and metastatic setting for mutational unselected patients, the benefit of cytotoxic therapy in *KRAS* mutant NSCLC has been an active area of research. A retrospective analysis conducted by Tsao and colleagues [19] looked at the prognostic effects of *p53* mutations or *RAS* mutations in a set of 450 patients with NSCLC who were randomized to observation or a platinum doublet in the adjuvant setting as part of the JBR10 study. In this group of patients, 117 patients harbored a *RAS* gene mutation (26 %). This study did not find that *KRAS* mutational status was a prognostic determinant of disease free survival. However, patients with *RAS* mutations who were given adjuvant chemotherapy did not appear significantly benefit from adjuvant therapy (HR of 0.95, 95 % CI 0.53–1.71, p = 0.87) compared to observation. In contrast, the wild-type *RAS* group, adjuvant chemotherapy did appear prolong survival significantly when compared to observation (HR of 0.69, 95 % CI 0.49–0.97, p = 0.03). An important caveat is that in this retrospective analysis, a test for interaction failed to find a significant interaction between chemotherapy and RAS mutational status (p = 0.29). Therefore, the authors concluded that *KRAS* mutation status was not a predictive factor for chemotherapy [19].

In order to obtain a more definitive answer, the Lung Adjuvant Cisplatin Evaluation Biomarker (LACE-Bio) collaborative group undertook a pooled analysis of *KRAS* mutant vs. wild type patients in four different trials to ascertain if *KRAS* mutational status had an effect on patients who received adjuvant chemotherapy. Data from 1543 patients (763 observation patients and 780 patients who received adjuvant chemotherapy) was compiled in a blinded fashion. In this analysis, 300 patients had KRAS mutations (19 %) including 275 codon 12 mutations, 24 codon 13 mutations and only 1 codon 14 mutation. The median follow-up time was 5.5 years and there were 754 deaths (49 %) among all patients [20].

This trial’s pooled results found that *KRAS* mutational status was weakly prognostic and did not show a significant predictive benefit from adjuvant chemotherapy. Between these studies, results were not significantly different with respect to disease free survival. The hazards ratio when comparing adjuvant chemotherapy with observation in the *KRAS* wild type patients was 0.89 (95 % CI, 0.76–1.05, p = 0.15). In comparison, the hazard ratio when comparing adjuvant chemotherapy with observation in the patients with *KRAS* mutations at codon 12 was 0.95 (95 % CI, 0.77–1.44, p = 0.77). Interestingly, there did appear to be small subset of KRAS mutant patients who failed to benefit from chemotherapy. Patients with codon 13 mutations seemed to have a detrimental effect from adjuvant chemotherapy (HR of 5.78, CI 2.06–16.22, p = 0.001). However, this was limited by the small sample size, as there were only 24 patients with codon 13 mutations but was statistically significant [20].

In the metastatic setting, several studies have looked at the predictive role of *KRAS* mutations in patients treated with chemotherapy. To date, the conclusions of these studies have been mixed with some studies finding that a *KRAS* mutation predicts for worse response and outcome while others have found no difference based on *KRAS* mutation status [21–24]. One of the first studies to examine this question retrospectively analyzed the prognostic and predictive value of *KRAS* mutations among 484 patients with *KRAS* and *EGFR* mutation information available. Of these patients, 8 % (39 patients) had *KRAS* mutations and 38 % (182) had *EGFR* mutations. In the multi-variate analysis, a *KRAS* mutation was a poor prognostic factor (hazard ratio = 2.6, 95 % CI 1.8–3.7). Patients with the *KRAS* mutation tended to do worse after gemcitabine-based or pemetrexed-based chemotherapy (RR 14 % mutation vs. 28 % in wild type) [21]. A second larger study looking at 204 patients with advanced non-squamous NSCLC that included 77 patients with *KRAS* mutant phenotype who had a significant inferior outcome with respect to disease response rate (0.04), disease control rate (0.05) and progression-free survival (p = 0.05) in patients being treated with first-line platinum-based chemotherapy regimens when compared to patients conferring *KRAS* wild-type and *EGFR* wild-type mutations [22]. However, a recent large study in 505 Caucasian patients that included 167 *KRAS* mutant patients failed to find a difference in either PFS or OS [24]. In summary, the *KRAS* mutation has been found to be predictive in some but not all studies and all these analyses are limited by their retrospective nature and diverse ethnic populations examined.

### Targeted approaches

Preclinical studies have demonstrated that localization of *KRAS* to the plasma membrane is essential for its function. One of the first efforts to block RAS signaling was with farnesyl transferase inhibitors. These drugs were designed to block farnesylation of RAS and therefore prevent its localization to the membrane and subsequent signaling. A phase II trial conducted examined the efficacy and pharmacodynamics of a farnesyl transferase inhibitor (R1155777) in 44 patients with NSCLC (mutation status unknown). Patients were pre-treated patients with Stage IIIB to Stage IV disease and were given R1155777 at 300 mg twice daily for 21 days out of a 28-day cycle. Unfortunately, there was no objective response seen in this study as seen in Table 1. Median survival was 7.7 months (95 % CI 6.5–10.5) and 7 patients had disease stabilization for 6 months (16 %, 95 % CI 6.5–10.5). Interestingly, 83 % of the patients had FTI inhibition in vivo although this did not correlate with objective response. Grade 4 toxicities observed in this study included neutropenia, anemia, and anorexia [25]. Although it is likely that a fraction of these patients would be *KRAS* mutant positive, it is unclear whether those patients with disease stabilization had *KRAS* mutant positive tumors. This class of drugs was never specifically tested in a defined *KRAS* mutant NSCLC population, in part due to the recognition that *KRAS* could be undergo an alternative modification which would allow it to localize to the membrane even in the absence of farnesylation. The failure of this agent dampened interest in further developed of any targeted agents for RAS for almost a decade [26].

**Table 1:** Selected Completed Clinical Trials in patients with *KRAS* mutant NSCLC

Agent	Mechanism of action	Number of patients	Setting	Study results	References
Farnesyl transferase inhibitor (R1155777)	Farnesyl transferase inhibitor	44 (mutation status unknown)	Second line and beyond	No objective responses Median survival of 7.7 months	[25]
Salirasib	Prevents localization of RAS to the plasma membrane	33 (30 *KRAS* mutant)	All-lines	No observed responses; 11 patients with stable disease at 10 weeks (7 previously treated and 4 previously untreated) Median overall survival not reached	[28]
Sorafenib	Tyrosine Kinase Inhibitor	27 *KRAS* mutant	Second line and beyond	*KRAS/BRAF* marker group who were treated with sorafenib had a 79 % disease control rate at 8 weeks	[30]
Sorafenib	Tyrosine Kinase Inhibitor	37 (11 *KRAS* mutant)	Second line and beyond	Disease control rate of 65 % Median PFS of 3.4 months Median OS of 11.6 months 20 patients had stable disease and 2 had partial response	[31]
MEK inhibitor	Selumetinib and Docetaxel	87 (All *KRAS* mutant)	Second line and beyond	Median OS of 9.4 months in the selumetinib group compared to 5.2 months in placebo Median PFS was 5.3 months in the selumetinib group and 2.1 months in placebo	[36]
MEK inhibitor	Trametinib	129 (All *KRAS* mutant)	Second line and beyond	Median OS was 8 months in trametinib arm and not reached docetaxel arm Median PFS 3 months in trametinib group and 2.75 months in the docetaxel group 10 partial responses in trametinib group and 5 in docetaxel group	[38]
CDK Inhibitor	LY2835219	49 (26 *KRAS* mutant)	Second line and beyond	Overall disease control rate of 51 %. In KRAS mutant NSCLC, DCR of 54 % versus 37 % in KRAS wild type. Median duration of SD was 5.6 months Median PFS was 2.1 months	[46]

A second potential RAS inhibitor was developed again with the goal of inhibiting localization to the plasma membrane. Salirasib (s-trans, trans-farnyesilthiosalicylic acid) was developed as a RAS farnesylcysteine mimetic which prevents attachment to the RAS membrane [27]. FTS successfully outcompetes RAS for binding to its escort protein that possesses putative farnesyl-binding domains that are necessary for its localization to the membrane. Preclinical data in vitro and in vivo demonstrated promising efficacy in *KRAS* mutant driven malignancies and therefore, Salirasib was tested in *KRAS* mutant NSCLC in the clinic [28].

In a phase I trial salirasib’s safety profile was evaluated in patients with advanced cancers and neurofibromatosis. Common side effects included diarrhea (occurred in 79 % of patients), as well as fatigue, nausea and vomiting. Of note, there was not a single patient in this trial with *KRAS* mutant advanced lung cancer [29]. Subsequently, Riely et al. [28] conducted a Phase II trial to determine the activity of salirasib in patients with advanced *KRAS* mutant lung cancer (Stage III–IV). A total of 33 patients were enrolled in the trial of which 30 had *KRAS* mutant NSCLC. The primary end point was rate of non-progression at 10 weeks and the secondary endpoints included RECIST response rate, duration of response, time to progression, and overall survival (OS).

The results from this trial were disappointing. No responses were observed. In patients who were previously treated with chemotherapy, 7 out of the 23 had stable disease at 10 weeks (30 %, 95 % CI 15–51 %). In the patients not previously treated with chemotherapy, 4 out of the 10 had stable disease at 10 weeks (40 %, 95 % CI 17–69 %). Of these 11 patients who had stable disease, the median time of stable disease was about 7 months. Overall, the median survival was 15 months in the pre-treated patients and was not reached (>9 months) in the untreated patients as seen in Table 1. This was at the cost of moderate toxicity of diarrhea, nausea and fatigue [28]. Although this trial’s results and toxicities were disappointing, it is significant that it was the first trial to prospectively examine a targeted therapy specifically in *KRAS* mutant NSCLC.

Kim et al. [30] subsequently published a trial in 2011 that was quite unique in that it is a prospective trial that mandated biopsies in patients with pre-treated lung cancer. This trial entitled biomarker-integrated approaches of targeted therapy for lung cancer elimination (BATTLE) enrolled 255 patients with pre-treated non-small cell lung cancer who were randomized into one of four arms based on biomarker analysis: erlotinib, vandetanib, erlotinib plus bexarotene, or sorafenib. Subsequently, patients were randomized in an adaptive manner into four predefined biomarker subgroups including *EGFR* mutation/copy number, *KRAS/BRAF* mutation, *VEG/VEGFR*-*2* expression, *RXRs/Cyclin D1* expression and *CCND1* copy number. In this study population, about 20 % had a *KRAS* mutation and another 15 % had *EGFR* mutations.

The results of this trial show an impressive benefit to patients with *KRAS* mutant genotype who were treated with sorafenib. Those with *KRAS/BRAF* marker group who were treated with sorafenib had a 79 % disease control rate at 8 weeks compared to 14 % in those just treated with erlotinib (p = 0.016). However, sorafenib produced the most drug toxicity causing 21 % treated dose reductions and causing 19 % discontinuation of the drug. The median overall survival was 8.8 months (95 % CI 6.3–10.6) and the median patient follow-up was 10.3 months. The 1-year survival was 35 %. There were no complete responses and only 9 partial responses. These findings are currently being followed up in the BATTLE-2 trial, which hopes to look at pre-specified biomarkers and then conduct prospective testing for biomarker signatures [30].

Despite the provocative findings above, it is unclear whether sorafenib will be an effective therapeutic strategy for *KRAS* mutant NSCLC. Another study conducted at the National Cancer Institute looked at sorafenib in mutationally defined populations as well. Thirty-seven patients with relapsed or recurrent NSCLC were enrolled in this trial and given sorafenib at 400 mg by mouth twice a day for a 28-day cycle. The median duration of treatment was 3 months. *KRAS* mutations occurred in 32 % of patients (11/34). However, *KRAS* or *EGFR* status showed no correlation with response (progression free survival (PFS) or OS). The disease control rate in the *KRAS* mutant group was 60 % and was 71 % in the *KRAS* wild-type group (p = 0.69). The disease control rate in the EGFR mutant group was 40 % and in the wild-type group was 69 % (p = 0.33). The conclusion made from this trial is that sorafenib inhibits progression of disease independent of *KRAS* mutational status with no statistical differences when looking at either PFS or OS [31]. Similar results were found in patients with *EGFR* mutations as well. This study is limited primarily by the small size but did have a varied population that is representative of the scope of patients seen in lung cancer clinics nationwide.

## Current therapeutic strategies in clinical trials for targeting *KRAS* mutant NSCLC

### MEK inhibitors

Two MEK inhibitors (MEKi), selumetinib and trametinib, have been explored as either monotherapy or in combination with cytotoxic agents to target *KRAS* mutant NSCLC. Selumetinib, was developed by AstraZeneca is an oral, selective non-ATP competitive inhibitor of MEK1/MEK2, which are key mediators of *KRAS* signaling (Fig. 1) [32]. Early phase studies showed that selumetinib monotherapy resulted in tumor response in patients with advanced cancer [33]. However, phase II studies with selumetinib monotherapy showed little clinical activity in patients with previously treated NSCLC including patients that had *KRAS* mutations [34]. Although monotherapy did not appear to be effective in the clinic, in vivo models looking at docetaxel in combination with selumetinib found that there was a synergistic effect that resulted in tumor growth inhibition and regression [17]. A subsequent phase I study in the setting of advanced cancers showed that the side effects of docetaxel and selumetinib were manageable [35].

Therefore a phase II trial was conducted to look at the combination of selumetinib and docetaxel in patients with *KRAS* mutant NSCLC. Participants in this study had stage III-IV NSCLC with *KRAS* mutant disease and had failure of treatment after first-line chemotherapy. The study accrued 87 patients who were randomized 1:1 to receive selumetinib and docetaxel or to receive placebo and docetaxel in a double blind fashion. Patients received docetaxel at 75 mg/m^2^ on day 1 every 21-day cycle and were expected to receive a total of 6 cycles. Patients also received either selumetinib capsules 75 mg twice a day or placebo capsules twice a day until disease progression or toxic effects of the drug. The primary end point of this study was overall survival and secondary endpoints were PFS, proportion of patients with objective response, duration of response, change in tumor size, 6-month PFS and safety [36].

Although the trend in overall survival was encouraging, the study failed to meet its primary endpoint. Median overall survival was 9.4 months (95 % CI 6.8–13.6) in the selumetinib group and 5.2 months (3.8–not calculable [NC]) in the placebo group (HR for death with selumetinib, 0.80, 80 % CI 0.56–1.14; one-sided p = 0.21) and hazards ratio were non-proportional (HR for death with selumetinib 0.8, 80 % CI 0.56-1.14, one sided p = 0.21). Median progression free survival was 5.3 months (95 % CI 4.6–6.4) in the selumetinib group and 2.1 months in the placebo group (95 % CI 1.4–3.7) with a HR for progression with selumetinib of 0.58 (80 % CI 0.42–0.79, one-sided p = 0.014) as seen in Table 1. No complete responses were observed. However, 16 patients in the selumetinib arm had partial response versus no patients in the placebo arm had a partial response thus a 37 % objective response for selumetinib [36]. Unfortunately, the utility of this regimen was limited by the higher frequency of adverse events in the selumetinib group. Febrile neutropenia occurred in 14 % of the selumetinib group and did not occur in the placebo group. Additionally, pneumonia occurred in 9 % of the selumetinib group and did not occur in the placebo group. [36].

This was the first prospective study to examine the efficacy of docetaxel in combination with selumetinib, a MEK1/2 inhibitor, in patients with pre-treated *KRAS* mutant Stage III-IV NSCLC Overall survival did not significantly improve however there were statistically significant improvements in progression-free survival and response rate. One major drawback of the study was the study size. Although more than 300 patients were screened for the study, only 87 were included in the study. The success of these drugs in combination was at the cost of adverse events including febrile neutropenia, pneumonia and neutropenia. Currently an ongoing phase III trial with mandated growth factor support is underway assessing the efficacy and safety of selumetinib and docetaxel in advanced or metastatic *KRAS* mutant NSCLC in the second line and beyond (NCT01933932).

A second MEKi, trametinib which is a selective allosteric inhibitor of MEK1/MEK2, has also been examined as a single agent in *KRAS* mutant NSCLC. The first trial of trametinib monotherapy in *KRAS*-mutant NSCLC patients showed 2/30 (2 %) partial responses and 16/30 (53 %) stable disease response [37]. In a subsequent phase II trial conducted by Blumenschein and colleagues [38], patients with *KRAS* mutant NSCLC who had previously been treated with a platinum regimen were randomized in a 2:1 fashion with trametinib (2 mg once daily) or docetaxel (75 mg/m^2^ IV every 3 weeks). There were one hundred and twenty-nine patients enrolled in this study in which 86 patients received trametinib and 43 received docetaxel. The primary end point analyzed was progression free survival. Progression free survival was 12 weeks in the trametinib group and 11 weeks in the docetaxel group (HR of 1.14; 95 % CI 0.75–1.75; p = 0.5197). This was not statistically significant or clinically meaningful. There were 10 (12 %) partial responses in the trametinib arm and 5 (12 %) in the docetaxel arm (p = 1.00). No patients had a complete response but 3 patients in the trametinib arm had tumor reduction of greater than 80 %. The overall survival was 8 months in the trametinib arm and was not reached in the docetaxel arm. The overall response rate was 12 % in the trametinib arm and 12 % in the docetaxel arm. Unfortunately, the trial ended prematurely due to interim analysis results that showed that the trial crossed the futility boundary after the 92 PFS events were analyzed. In the trametinib arm, all patients had some type of adverse event (mostly grade 1 or 2). The most common adverse effects were rash, diarrhea, hypertension, nausea, dyspnea and fatigue and 5 fatal events were reported. In the docetaxel group, the most common adverse event was neutropenia and no fatal events were noted. In this study, trametinib did not show superiority to docetaxel as a single agent. However, the authors suggested that similarly to selumetinib, there is strong rationale to trametinib with other chemotherapy agents, biologics or radiation as a potent therapy.

### Focal adhesion kinase (FAK) inhibitors

Other efforts to target *KRAS* mutant NSCLC have focused on inhibition of the focal adhesion kinase (FAK). The extracellular signal-regulated kinase ERK/RHOA/(FAK) network is unregulated in patients with lung cancer. Furthermore, the KRAS-RHOA-FAK signaling pathway appears to be critical for *KRAS* mutant tumorigenesis in the subset of lung cancers that have lost either *TP53* or the *CDKN2A* loci that encodes p16 and ARF proteins. Furthermore, inhibition of FAK causes tumor regression in mice with mutant *KRAS* and *Cdkn2a* deficiency [39]. These studies served as the preclinical rationale for testing the FAK inhibitor, Defactinib (VS-6063) in *KRAS* mutant NSCLC. To test the efficacy of FAK inhibition in *KRAS* mutant NSCLC with distinct mutation cohorts (A: *TP53*, *CDKN2A* wild type, B: *TP53* wild type, *CDKN2A* altered, C: *TP53* mutant, *CDKN2A* wild type, and D: *TP53* mutant, *CDKN2A* altered), a phase II multi-center study is currently underway in patients with *KRAS* mutant NSCLC who have already received one platinum doublet regimen. The trial has completed accrual and the data analysis is underway [40] as seen in Table 2. This trial represents the first effort to test the efficacy of a targeted therapy in a molecular defined subset of *KRAS* mutant NSCLC and may serve as a model for future trials.

**Table 2:** Selected Ongoing Clinical Trials in patients with *KRAS* mutant NSCLC

Agent(s)	Mechanism of action	Phase	Setting	Sponsors	Primary endpoint	Clinical Trial Number
MEK162 + erlotinib	MEK inhibitor	I/IB	Second line and beyond	H. Lee Moffitt Cancer Center and Research Institute Novartis	MTD and PFS	NCT01859026
Selumetinib + Docetaxel vs. Docetaxel	MEK inhibitor	III	Second line	AstraZeneca	PFS	NCT01933932
Momelotinib alone and in combination with Trametinib	MEK inhibitor	Ib	Second line and beyond	Gilead Sciences	DLT and DCR at 8 weeks	NCT02258607
Abemaciclib vs. Eroltinib	CDK inhibitor	III	Second line and beyond	Eli Lilly	PFS and OS	NCT02152631
Palbociclib (PD-0332991) + PD 0325901	CDK 4/6 inhibitor + MEK inhibitor	I/II	First line and beyond	Dana-Farber Cancer Institute	MTD and RP2D	NCT02022982
Retaspimycin (IPI-504) + EverolimusGI-4000	Heat Shock Protein 90 Inhibitor	Ib/II	Second line and beyond	Infinity Pharmaceuticals, Inc.	ORR	NCT01427946
Defactinib (VS-6063)	FAK inhibitor	II	Second line and beyond	Verastem, Inc	PFS12 in mutational defined cohorts	NCT01951690

### Cyclin-dependent kinase (CDK) inhibitors

Another potential set of targets that may be successfully targeted in *KRAS* mutant NSCLC are the cyclin-dependent kinases (CDKs) which are critical regulatory enzymes that drive the cell cycle. Many of the key concepts of the biology of CDK were learned greater than 20 years ago through the study of yeast. Proliferation requires the activation and formation of Cyclin/CDK4 or CDK6 complexes. Active Cyclin/CDK4/6 complexes phosphorylate the critical tumor suppressor, RB1 (Retinoblastoma 1) leading to gene expression and progression through the cell cycle. Additionally, CDK4 and CDK6 lead to increase levels of expression and stability of E-type and A-type cyclins and activation of CDK2 leading to entry into S phase and DNA replication [41]. Interestingly, preclinical data both in vitro and in vivo suggest that CDK function is critical for KRAS tumorigenesis and that inhibition of CDK in *KRAS* mutant NSCLC leads to potent synthetic lethality [42].

Translating the knowledge that the cyclin dependent kinase pathway is a potential therapeutic target into a successful clinical drug has been challenging in various cancer subtypes. Flavopiridol was the first drug CDK inhibitor that was extensively investigated with over 60 clinical trials between 1998 and 2014. Unfortunately, flavopiridol had low levels of activity seen in Phase II clinical trials in several solid tumor types. Roscovitine is another CDK inhibitor that was evaluated in patients with NSCLC. The APPRAISE trial is a Phase II that randomized NSCLC patients to roscovitine versus best supportive care. The trial terminated after 187 patients were enrolled. The results were not published but were disappointing as there was no impact on progression free survival [41].

Recently, a novel CDK inhibitor, palbociclib has been demonstrated to have significant clinical activity in estrogen receptor positive (ER+) breast cancer patients which is likely due to the key role the CDK4/6-RB1-E2F plays in this breast cancer subtype. The PALOMA-1/TRIO-18 was a Phase II clinical study of 165 women with advanced ER+ breast cancer. This study compared the aromatase inhibitor letrozole alone versus the combination of letrozole plus the CDK4/6i, palbociclib and showed that the group that received the combination therapy had a significant improvement in median progression-free survival when compared with letrozole alone (20.2 months compared with 10.2 months; HR of 0.448; 95 % CI 0.319–0.748; p = 0.0004). Additionally, the overall survival analysis showed that there was a trend of favoring the letrozole plus palbociclib combination (37.5 vs. 33.3 months respectively, HR of 0.813, p = 0.2105) [43]. This lead to the FDA granting accelerated approval to palbociclib for use in combination with letrozole for the treatment of postmenopausal women in the first line metastatic setting in ER +, human epidermal growth factor receptor 2 (HER2)-negative breast cancer [44] and increased interest in examining this agent in other solid tumors including lung.

Efforts to use palbociclib in patients with NSCLC are currently in clinical trials, however, palbociclib has not been specifically examined in *KRAS* mutant NSCLC. In a Phase II study by Gopolan et al. [45], 19 previously treated patients with recurrent or metastatic NSCLC and negative for p16 expression by immunohistochemistry were given palbociclib at 125 mg daily with the primary endpoint of response rate. Of the 16 evaluable patients who received at least 1 month of therapy there were no responses. Eight patients had stable disease and the remaining eight patients had progressive disease within 8 weeks of treatment. The median progression free survival was 12.5 weeks. Grade 3 toxicities included thrombocytopenia and neutropenia that was seen in three patients. Another patient experienced rhabdomyolysis and transaminitis when taken together with high dose simvastatin. The drug was otherwise well tolerated.

Another CDK inhibitor LY2835219 has shown great activity specifically in *KRAS* mutant xenografts and is currently being tested in *KRAS* mutant NSCLC. An initial Phase I trial was conducted in a 49 patient cohort of progressive or metastatic NSCLC patients who had had an average of 4 lines of treatment. In this cohort of patients, 26 were *KRAS* mutant and 19 *KRAS* wild type. Patients were given an oral pill of LY2835219 for a 28-day cycle. The drug was relatively well tolerated with 41 % of patients reaching at least 4 cycles with only 2 % of the study cohort suffering from grade 3 toxicities including diarrhea and nausea, and 2 % suffering from fatigue, vomiting and anemia. Consistent with preclinical studies, the study drug was more effective in patients with *KRAS* mutant NSCLC. The disease control rate was 54 % in *KRAS* mutant patients [46]. Given these promising findings, a phase III trial is being conducted using Abemaciclib (LY2835219) plus best supportive care versus erlotinib plus best supportive care in patients with *KRAS* mutant pretreated NSCLC. This study is currently underway and recruiting patients. (NCT02152631).

### Heat shock protein 90 (Hsp90) inhibitors

Hsp90 is part of the class of molecular chaperone proteins that plays a central role in the assembly of multi-protein chaperone complexes and regulates folding, stability and function of many client proteins that are oncogenic drivers of lung adenocarcinoma subsets such as mutant EGFR, wild-type CRAF, mutant BRAF, wild-type and mutant HER2, and EML4-ALK fusion protein positive NSCLC. The inhibition of Hsp90 depletes these kinases from cancer cells and disrupts signaling pathways that are critical for proliferation and survival, and thus are an exciting therapeutic target [47].

Since Hsp90 inhibitors are able to inhibit multiple downstream signaling pathways of mutant *KRAS*, there has been significant interest in testing Hsp90i in *KRAS* mutant NSCLC. In a trial by Socinski and colleagues in 2013 [48], ganetespib was given to 99 patients with a median of 2 prior systemic therapies. Patients were divided into three cohorts, including mutant *EGFR*, mutant *KRAS* and wild type for EGFR and *KRAS*. Patients were given 200 mg/m2 ganetespib intravenously once weekly for 3 weeks until disease progression. At 16 weeks, the EGFR mutant patients had a 13.3 % progression free survival, the *KRAS* mutant patients had a 5.9 % progression free survival and those that were *KRAS/EGFR* wild type had a 19.7 % progression free survival. Interestingly, four patients who harbored the anaplastic lymphoma kinase (ALK) gene rearrangement had a partial response and there appears to be significant single agent activity in this patient population. Eight patients (8.1 %) experienced treatment related serious advents of which 2 resulted in death (cardiac arrest and renal failure). The most common adverse events included diarrhea, fatigue, nausea and anorexia. In summary, single agent Hsp90i have failed to demonstrate significant activity in *KRAS* mutant NSCLC.

Since preclinical studies demonstrated a synergistic effect of taxanes in combination with ganetespib, this combination has been examined in a completed Phase II where PFS in *KRAS* mutant patients was a co-primary endpoint and an ongoing Phase III trial. The GALAXY-1 trial looked at ganetespib in combination with docetaxel in pretreated patients with NSCLC. Patients were given docetaxel (at 75 mg/m^2^ on day 1) alone or in combination with ganetespib (at 150 mg/m^2^ on days 1 and 15) of a 21-day cycle. Patients were randomized in a 1:1 fashion between docetaxel and docetaxel and ganetespib therapy. In this trial, 385 patients were initially enrolled but it was found early on that the combination therapy resulted in hemoptysis and lack of efficacy in those that did not have adenocarcinoma. Subsequently, only patients with adenocarcinoma were enrolled in this trial. There was a trend in favor of the combination therapy in the adenocarcinoma treated arm with respect to progression free survival (n = 253, HR of 0.82, p = 0.0784) and overall survival (HR of 0.84, p = 0.1139). The most common grade 3 adverse events in the combination arm included neutropenia, leukopenia, anemia and neutropenic fever. Grade 1 or 2 diarrhea was common in the combination arm about 48 h after the infusion. For the patients with *KRAS* mutations (89 patients), the combination therapy did not result in improved progression free survival (combination median 3.9 months vs. control of 3.0 months, HR of 1.1) or overall survival (combination median 7.6 months versus control of 6.4 months with HR of 1.23). One explanation regarding the lack of efficacy in the *KRAS* mutant population is that every 2 weeks dosing of ganetespib was not sufficient to suppress the KRAS pathway. Additionally, it was observed that those with advanced disease defined as advanced disease greater than 6 months before study entry seemed to do better with the ganetespib and docetaxel combination therapy with regards to progression free survival (n = 177, adjusted HR of 0.74, p = 0.0417) and overall survival (adjusted HR = 0.69, p = 0.0191) [49]. Currently, the next phase of the study called GALAXY-2 is currently enrolling patients and will definitely determine if there is any benefit in the *KRAS* mutant NSCLC patient population. This Phase III study is looking at patients with pretreated advanced NSCLC (diagnosis greater than 6 months prior) and randomizing in a 1:1 fashion with docetaxel and the ganetespib and docetaxel combination (NCT01798485). Finally, preclinical studies have suggested that *KRAS* mutant NSCLC may be more sensitive combinations of Hsp90i and other pathway inhibitors such as MTOR inhibitors [50, 51]. Results of phase Ib/II trial looking at the combination of the HSP90i, Retaspimycin (IPI-504) and TORC1 inhibitor, Everolimus is still pending as seen in Table 2.

## Future directions

In addition to the current strategies being tested in the clinic to target *KRAS* mutant NSCLC, several potential promising preclinical agents are in development. One class of promising agents is the direct RAS inhibitors including those that directly target the *KRAS* mutant protein. As oncogenic mutations inhibit GTP hydrolysis and therefore drives the activation of RAS; direct inhibition of *KRAS* proteins would have great clinical significance. Ostrem, Peters and their colleagues [52] reported the development of a small molecule that irreversibly binds to the *KRAS* mutant molecule (G12C). The molecule targets the mutant cysteine amino acid which is only present in *KRAS*-mutant proteins and therefore does not interfere with wild-type *KRAS* proteins. Binding of these inhibitors to *KRAS* at the switch-I and switch-II regions of the molecule shifts the preference to GDP rather than GTP impairing RAF binding. This study suggests that it may be possible to directly inhibit RAS proteins and target mutant proteins with no affect on wild-type proteins. Currently, these inhibitors are pre-clinical compounds; however, there is significant increase in the development of second generation compounds that could be tested in a phase I or II trials. Others groups are actively pursuing strategies to develop compounds that bind either RAS-GDP or RAS-GTP isoforms and prevent critical intra-molecular interactions with key RAS signaling partners [53]. In addition, there has been a reemergence of interest in preventing RAS localization to cellular membranes [54]. Furthermore, multiple preclinical studies have focused on identifying synthetic lethal interactions with *KRAS* mutations; however, the majority of these synthetic lethal interactions have not been reproducible [55]. This may be in part to the context dependence nature of these interactions. These studies lead in part to the realization that responses to agents may depend on presence or/absence of genetic modifiers such as TP53 mutations, CDKN2A alterations or STK11/LKB1 alterations [17, 39]. As discussed above, there is already one example of a phase II trial of a FAK inhibitor which presorted *KRAS* mutant patients into four cohorts based on the co-occurrence of TP53 mutations or CDKN2A mutations [40]. This is likely the first of many trials that will go beyond just selecting patients based solely on the presence of a *KRAS* mutation and hopefully lead to more effective treatments for genetically defined subsets of *KRAS* mutant NSCLC.

## Conclusions

In conclusion, the *KRAS* mutation is the most common oncogene driver mutation in patients with NSCLC and confers a poor prognosis in the metastatic setting making it an important target for drug development. This mutation that was discovered more than 3 decades ago result in the loss of GTPase activity making the onco-protein constitutively active and leading to activation of the RAF-MEK-ERK (MAPK) signaling pathway. This pathway is quite complex and multiple strategies have been proposed to target this mutation. To date, an effective agent to *KRAS* mutant NSCLC remains elusive.

In the last 5 years, there has been an impressive amount of drug development that has resulted in better understanding of the pathway. But unlike *ALK* and *EGFR* mutations, there is still no targeted therapy available for patients with *KRAS* mutations. Currently, there are promising strategies in clinical trials. We feel that the most promising of these agents are the MEKi agents (Trametinib and Selumetinib in combination with chemotherapy), CDK inhibitors, and hopefully direct *KRAS* inhibitors. We hope that these strategies result in successful drug development and provide an era of personalized medicine for this common mutation.

## Abbreviations

NSCLC: non small cell lung cancer
*KRAS* mutation: Kristen Rat Sarcoma virus mutation
*EGFR* mutation: epidermal growth factor receptor (EGFR) mutation
*ALK* mutation: anaplastic lymphoma kinase (ALK) mutation
FAK inhibitor: focal adhesion kinase inhibitor
CDK inhibitor: cyclin dependent kinase inhibitor
Hsp90 inhibitor: heat shock protein 90 inhibitor
